# Global collaborative healthcare: assessing the resource requirements at a leading Academic Medical Center

**DOI:** 10.1186/s12992-017-0298-5

**Published:** 2017-09-20

**Authors:** Nicole J. Rosson, Heitham T. Hassoun

**Affiliations:** 1 Johns Hopkins Medicine International, Baltimore, MD USA; 2 Department of Surgery, Johns Hopkins University School of Medicine, 1300 Thames Street, Suite 200, Baltimore, MD 21231 USA

**Keywords:** Globalization, Global collaborative health, Resource utilization academic medical centers

## Abstract

**Introduction:**

Academic Medical Centers (“AMCs”) have served as a hub of the United States (“US”) health system and represented the state-of-the art in American health care for well over a century. Currently, the global healthcare market is both massive and expanding and is being altered by the unprecedented impact of technological advances and globalization. This provides AMCs a platform to enter into trans-national collaborative partnerships with healthcare organizations around the world, thus providing a means to deliver on its promise globally while also expanding and diversifying its resources. A number of leading US AMCs have engaged in global collaborative healthcare, employing different models based on services offered, global distribution, and inclination to assume risk. Engaging in these collaborations requires significant effort from across the health system, and an understanding of the resources required is paramount for effective delivery and to avoid overextension and diversion from the primary mission of these organizations.

The goal of this paper is to discuss the role of US AMCs in this current global healthcare landscape and to also investigate our institutional faculty and staff resource requirements to support the operating model.

**Methodology:**

We extracted and retrospectively analyzed data from the JHI Global Services database for a 3-year period (Jan, 2013–Dec, 2015) to determine total utilization (hours and full time equivalent (FTE)), utilization by profession, and clinical and non-clinical areas of expertise.

**Results:**

JHI utilized on average 21,940 h annually, or 10.55 FTEs of faculty and staff subject matter experts. The majority of the hours are for work performed by physician faculty members from 23 departments within the School of Medicine, representing 77% percent or on average 16,894 h annually. Clinical and allied health departments had an average annual utilization of 17,642 h or 7.8 FTEs, while non-clinical departments, schools and institutes averaged 4298 h or 1.9 FTEs, representing 80.4% and 19.6% respectively.

**Conclusion:**

We found that significant human resources are required within a broad range of AMC subject matter expertise across multiple disciplines, and that with adequate forecasting AMCs can successfully engage in these collaborations while continuing to fulfill their core mission.

## Background

Academic Medical Centers (“AMCs”) have played a vital role in healthcare in the United States (“US”). They train thousands of the brightest physicians, nurses and allied health professionals on an annual basis and historically have been at the forefront of medical research, where new treatments and cures have been discovered [1]. In recent years, AMCs have faced numerous challenges at home including healthcare reform, regulatory changes, and declining reimbursement [2]. The era of “cost unconscious” third party payment for care is being transformed into a value-based system—growing pains are expected for years to come [3, 4]. These challenges have tested the ability of AMCs to continue to deliver on their mission and have required them to identify cost savings or revenue generating initiatives, including ways to maximize patient volumes and patient services revenue by targeting self-pay, out of state or international patients.

For decades, patients with the means to do so, or with government sponsorship, have traveled to receive care at premier healthcare institutions in Europe and the US, often times seeking out cutting edge care not available or readily accessible in their home country. These patients and their families present with a unique set of needs and challenges including but not limited to language, culture, and logistics [5]. As patient volumes increased so did the realization of the positive impact on the hospitals’ bottom lines. AMCs developed international patient services, including dedicated offices to manage these patients and endeavoring to maintain or increase patient volumes [6]. These patient care activities served as an entry point for AMCs to further develop existing trans-national relationships and to participate in global collaborative healthcare, offering consulting services, managing facilities abroad as well as establishing joint ventures and wholly-owned entities. Johns Hopkins Medicine International (“JHI”) has been at the forefront of global collaborative healthcare, but the model remains under development, evaluation, and refinement [7]. For some institutions there is a long-standing concern about the potential inability to successfully engage in international activities and meet client expectations, as well as the potential to drain resources and divert the focus from the AMCs’ core mission at home. This is certainly a potential risk if there is not sufficient redundancy or elasticity in an organization’s bandwidth. It can be argued that the positives, including the ability to improve the health of millions and the fruits borne from expanding collaborative opportunities into the realm of research or education, for example, far outweigh the tangible negatives [8]. The focus of this article will be a review of the operating model for global collaborative activities at leading US AMCs with an eye towards the resources required to support the international portfolio of activities and engagements.

## Global healthcare landscape

Globalization has impacted nearly every aspect of modern life [9]. From a healthcare perspective, patients have long traveled for complex clinical care and innovative treatments that are not available in their home country. In addition, healthcare professionals, primarily physicians and nurses, have a history of moving from one country to another for education and training purposes or seeking employment opportunities. While healthcare may have lagged behind other industries, it too has been impacted by a convergence of forces as a result of globalization [10]. Emerging economies are investing in healthcare infrastructure in order to expand access and improve quality and services. There has been an increase in the number of countries offering government sponsored universal healthcare coverage and requiring employers to provide coverage to employees [11]. As demand is increasing, there is more local privatization and increased competition with providers looking to differentiate themselves through international accreditation, high quality and low cost care, and hi-tech hi-touch services [6]. These developments have created favorable conditions and opportunities for US AMCs to collaborate on a global scale.

## Model for global collaborations

US AMCs have employed different models to engage in global collaborative healthcare. In the late 1990’s Johns Hopkins Medicine (“JHM”) formed a limited liability corporation to engage in these opportunities, creating Johns Hopkins Medicine International (“JHI”). JHI is the institutional platform that facilitates the global expansion of the JHM mission: to improve the health of the community and the world by setting the standard of excellence in medical education, research, and clinical care. JHI operates from its hub in Baltimore, Maryland and places executive, expatriate teams in the hospitals it manages or jointly owns.

**Fig. 1:**
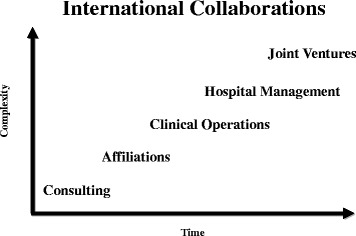
Depiction of Johns Hopkins Medicine International’s (JHI) structured collaborations which have increased in complexity over time

Over time, the size and scope of JHI’s engagements have increased, from short term consulting engagements to projects of greater complexity and resource requirements, such as affiliations, clinical operations, and hospital management agreements (Fig. 1). Consulting agreements can be short-term or long-term with clear objectives for providing expert knowledge and serving in an advisory capacity. Affiliations are more formal long term agreements, typically for 10 years, which involve a licensing agreement for the use of the Hopkins name. Clinical Operations agreements are solely focused on the enhancement and management of a particular clinical service line, which may be a component within a larger organization. Hospital management agreements are typically long term engagements and include the use of the Hopkins name as well as the placement of on-site Johns Hopkins International executives to manage the day to day operations of the hospital. Joint ventures involve the joining of forces with another organization, each contributing assets that result in the formation of a new legal entity, with joint ownership and risk sharing.

**Fig. 2:**
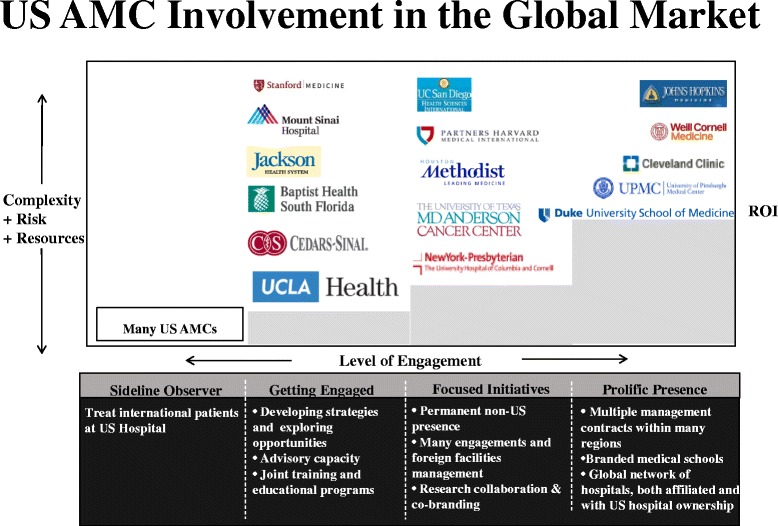
The levels of engagement of United States (US) academic medical centers (AMC) in the global healthcare market. With increased involvement, there is increasing complexity and risk attached to these collaborations as well as return on investment (ROI)

Other leading US AMCs have chosen different levels of engagement and employ different models for global collaborations (Fig. 2). Regardless of the model, the complexity, investment of time and risk increase depending on the service offerings ranging from consulting or advisory services to joint education or training programs, hospital management or ownership of healthcare facilities outside the US [6]. Many AMCs treat international patients and have gone on to develop services and offices to accommodate international patients who travel to receive care at their centers. The best example of such a model is the Mayo Clinic which has excelled at focusing on clinical care and patient services offered at its US-based hospitals. Other AMCs have expanded beyond direct patient care at home. For example, the University of Pittsburgh Medical Center (“UPMC”) has had a significant international presence, uniquely having developed long-term collaborations in Europe, focusing on specific clinical services management such as oncology in Ireland and transplant in Italy [12]. Based in Texas, Houston Methodist Hospital has a long-standing history of treating international patients seeking care under Dr. Michael E. DeBakey and his team of cardiac surgeons. Their international arm has expanded over the years, establishing offices in the Kingdom of Saudi Arabia ("KSA") and the United Arab Emirates (“UAE”) to manage and oversee their projects, thus demonstrating significant institutional commitment. Cleveland Clinic has long focused on the delivery of clinical care, using hospital management and ownership models as the vehicle to do so, with deployed clinical teams. Cleveland Clinic Abu Dhabi represents one of the world’s largest healthcare investments in Abu Dhabi, modeled as an extension of US-based Cleveland Clinic’s model of care at an estimated $2 billion dollars for its 4.4 million square foot facility [13]. Cleveland Clinic is currently developing their second facility outside of the US in London, England. Like healthcare, medical education is undergoing significant globalization, providing additional opportunities for AMCs [14]. Both Duke University, in its partnership with National University of Singapore Graduate Medical School and Weill Cornell Medical College working with the Qatar Foundation, have established US style medical schools in the respective countries, entering into historic relationships that require dedicated commitment and prolific presence [15].

## JHI Global Services

JHI is primarily focused on two business lines, Patient Services and Global Services, with an executive management that oversees both. Patient Services facilitates all aspects of care for patients who travel to JHM hospitals in the US. The focus of this paper is on Global Services which is responsible for exploring new business opportunities, working with government organizations, academic institutions, and health care providers to reinvigorate or develop radically new standards and models of patient care, medical education, and research. In recent years, JHI has expanded into the realm of integrated healthcare system development, large-scale knowledge transfer and education initiatives, and jointly owned and managed facilities outside the United States, such as its joint venture with Saudi Aramco in KSA, Johns Hopkins Aramco Healthcare. Since its inception, JHI has worked in over 50 countries in Asia, North and South America, Europe, the Middle East, and Africa. Currently there are 16 active projects in 14 countries.

JHI’s affiliate, managed or owned facilities (referred to as partners) are expected to be managed with high standards of care and with a goal of moving toward the standards associated with JHM. As part of its legal agreements, all affiliated or managed hospitals are required to demonstrate a commitment to the *Clinical Requirements for Hospitals Affiliated with Johns Hopkins Medicine International.*

### Clinical requirements for affiliation

A fundamental principle that guides the day-to-day activities of Hopkins’ institutions is to provide the best medical care possible in a safe and caring environment. This work is the basis for the *Clinical Requirements for Hospitals Affiliated with Johns Hopkins Medicine International.* The primary function of the *Requirements* is to allow for the monitoring of the quality of care delivered at the hospital to ensure the quality is consistent with the image and reputation of JHM. The areas of focus are 1) clinical quality, 2) risk management, 3) patient safety, and 4) international accreditation, i.e. Joint Commission International ("JCI").

As part of the *Requirements,* clinical key performance indicators ("KPIs"), specific clinical and quality monitoring measures, are developed annually in a collaborative effort with JHI, JHM and the partner. The KPIs are submitted to JHI on a monthly basis and are reviewed quarterly at the JHI Quality Forum. The *Clinical Requirements* also establish a common baseline for partner hospitals for becoming accredited by JCI, or an equivalent accreditation agency.

### Clinical, medical and project teams

In support of the *Clinical Requirements*, and with the increasing complexity of JHI’s engagements and growth in volume and geographical scope, the Nursing and Clinical Quality function grew beyond accreditation and clinical quality to include a Nursing and Clinical Affairs leadership position and several key positions in risk management, regulatory affairs, analytics, and nurse consultants. In addition, the JHI physician leadership developed to include both JHI project medical directors and a Global Medical Director, whose primary function is to provide medical leadership and direction for international projects, and to serve as the conduit between three primary entities: JHI leadership, JHM clinical departments, and our global partners. The nursing and physician teams work to ensure that Johns Hopkins standards of excellence and best practices are implemented across our global network.

Global Services is further organized and supported by regional project teams. These regional teams pursue new business development opportunities and provide project management support for existing partners, ultimately endeavoring to meet the needs of partners and ensuring contractual deliverables are met. The project team serves as liaisons between partners and other components of JHM and Johns Hopkins University, institutions who provide expert assistance to our international partners. The project teams provide project management specific administrative work in support of the subject matter experts ("SME"). To facilitate this work, many partners have defined knowledge transfer programs that serve as a roadmap and conduit for the development and enhancement of administrative, operational and clinical programs. The delivery of knowledge transfer comes in many forms including assessments, development of plans and implementation for new or enhancement of clinical or administrative services, clinical rotations, administrative and clinical observerships, workshops, courses, and conferences provided at either partner facilities or Johns Hopkins entities in the US. At present, in addition to the executive management team, there are approximately 77 full time equivalents ("FTE") within Global Services and JHI core functions that support our global collaborations.

## Resource requirements for global collaborations

JHI engages a wide range of SMEs from the entities that comprise JHM and Johns Hopkins University. These experts provide assistance to JHI’s partner institutions to shape strategy, be catalysts for change, and create sustainable improvements in care delivery. There are concerns that by participation in these activities, attention and resources are diverted from the core JHM mission in the US. Over time, a resource forecasting component has been included for new projects to ensure that there is an understanding of the resources required to successfully fulfill obligations and that there is leadership support across the organization. We sought to measure the human resource and subject matter expertise requirements to support the current JHI portfolio of global collaborations.

### Study design: work orders and downstreaming

In order to facilitate and monitor the use of SMEs and resources from Johns Hopkins, JHI develops work orders, which are standardized contractual agreements that define the services to be provided, including objectives and deliverables, timelines, time commitment, payment and additional terms and responsibilities. We extracted and retrospectively analyzed data from the JHI Global Services database for a 3-year period (January 2013–December 2015) to determine total utilization (hours and FTE), utilization by profession, and clinical and non-clinical areas of expertise.

### Data sources

These work orders are executed and retained in the JHI Global Services database. Global Services maintains this database, which interfaces with financial systems, to facilitate the work of the regional project teams and core support functions. These function include invoicing, generation of project profit loss statements and payments to JHM entities and JHU schools and departments, which is referred to as downstream. Requests for downstream payments are entered by JHI Global Services staff and processed by Finance upon completion of the defined deliverable. Data was extracted from the JHI Global Services Database based on a query for all downstream payments that were processed upon completion of requested work for 36 months between January 2013 and December 2015.

### Data analysis

Data was sorted and analyzed by a number of variables: 1) Total Utilization (hours and FTE), 2) Break out by professional category, 3) Clinical Departments, Schools, Institutes and Non-Clinical Departments and 4) Project Type and Region. For purposes of this analysis, 1 FTE = 2080 h and the standard JHI Global Services consulting rates for physicians, nurses, administrators and other staff were used for the conversion. This includes all work performed on behalf of JHI Global Services and excludes work performed related to direct patient care provided overseas or in the US.

### Results for faculty and staff utilization

**Fig. 3:**
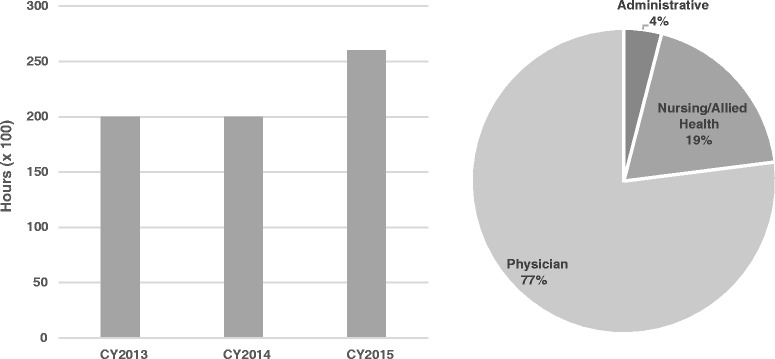
**a** Total faculty and staff utilization by calendar year (CY) for 2013–2015. **b** Percent effort based on areas by professional category

During this 3 year time period, JHI utilized on average 21,940 h annually, or 10.55 FTEs of faculty and staff SME. The majority of the hours are for work performed by physician faculty members from the School of Medicine, representing 77% percent or on average 16,894 h annually. The remaining 23% (5046 h) is a combination of nursing, allied health professionals and non-clinical staff. Nursing and allied health professionals represent 19% or 4168 h and non-clinical staff at 4% percent or 878 h (Fig. 3).

**Fig. 4:**
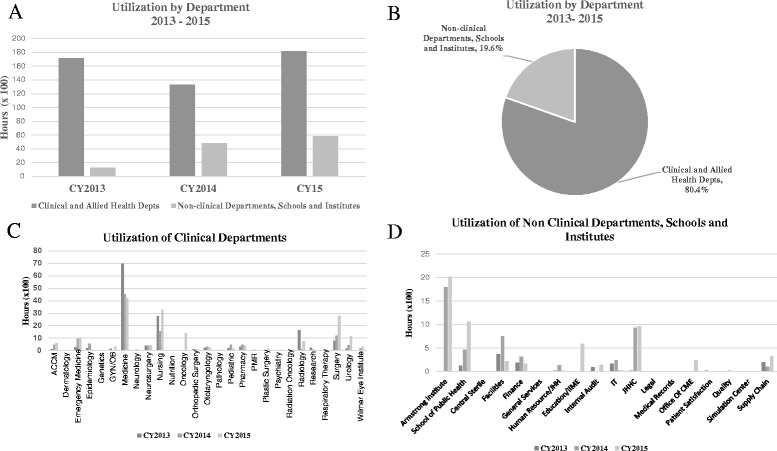
**a** Annual utilization trends of clinical and allied health departments versus non-clinical departments, schools and institutes for 2013–2105. **b** Average annual percent effort clinical and allied health departments versus non-clinical departments, schools and institutes. **c-d** Break out of annual utilization of clinical and allied health department utilization (**c**) and non-clinical departments, schools and institutes (**d**) for 2013–2015

When looking at the breakdown of clinical and allied health departments versus non-clinical departments, such as Finance, Facilities, Supply Chain and Information Technology, schools and institutes, the following trends were found. Clinical and allied health departments had an average annual utilization of 17,642 h or 7.8 FTEs, while non clinical departments, schools and institutes averaged 4298 h or 1.9 FTEs, representing 80.4% and 19.6% respectively. Within the clinical and allied health departments, Medicine and Nursing had the highest utilization, with an annual average 5239 h and 2537 respectively followed by Surgery and Research with 1603 h and 1309 h respectively. Within administrative departments, schools, and institutes, the Armstrong Institute for Quality and Patient Safety had the highest utilization with 1914 h annually (Fig. 4).

**Fig. 5:**
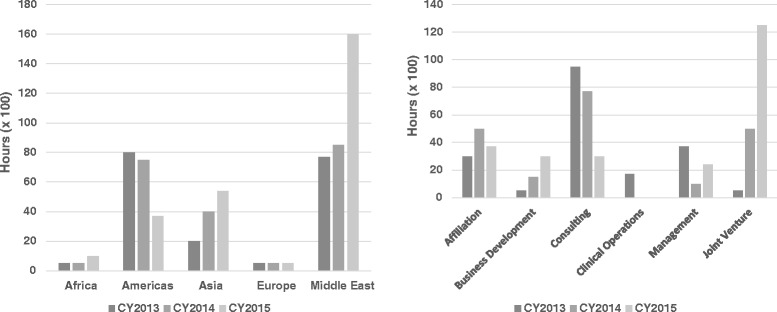
**a-b** Annual faculty and staff utilization by region for 2013–2015 (**a**) and project type (**b**) for 2013–2015

In terms of regional distribution, the Middle East averages the highest average utilization with 11,076 h (50.5%), followed by, the Americas, which compromises, North America, South America and the Caribbean with 6470 h (29.5%), and Asia with 3846 h (17.5%). In terms of project type, there was relatively even distribution across the spectrum of projects, but joint ventures and consulting required the highest average utilization with consulting utilizing 6939 h (31.9%) and joint ventures utilizing 6367 h (29.2%) respectively (Fig. 5).

### Discussion

US AMCs have long played a critical role in healthcare, with their innovation, discovery and academic pursuits transforming the standard of care at home and abroad. Currently, the global healthcare market is rapidly developing, providing a platform for AMCs to enter into collaborative partnerships with international healthcare organizations around the world, and providing a means for AMCs to deliver on its promise globally while also expanding and diversifying resources helping to continue to fulfill its mission in the US.

Our findings document that a broad range of subject matter expertise is required to engage in global collaborative healthcare. In evaluating the resources required to support the global portfolio of projects at a leading US AMC, we found that an average 21,940 h of subject matter expertise was required. 77% of total hours required physician faculty expertise and clinical and allied health departments had an average annual utilization of 17,642 h or 7.8 FTEs. The department with the highest utilization was Medicine.

Our evaluation of resource requirement has two limitations in that it does not necessarily reflect the true demand for select specialty expertise as the JHI model does not proactively seek projects or engagements within defined specialties and is therefore generally reactive as it works to meet the needs of existing partners.

## Conclusion

AMCs have long played a critical role in healthcare, with their innovation, discovery and academic pursuits transforming the standard of care in the US and abroad. Currently, the global healthcare market is rapidly developing, providing a platform for AMCs to enter into collaborative partnerships with international healthcare organizations around the world, providing a means for AMCs to deliver on its promise globally while also expanding and diversifying resources helping to continue to fulfill its mission in the US.

While many AMCs provide patient care services for international patients, a smaller, select group have opted to capitalize on the globalization of healthcare and engage in global collaborations across the continuum from short-term consulting agreements to joint ventures and owned facilities. These AMCs have increased the complexity of their collaborations, and with that assumed the associated higher risk and upside of a higher return on investment. All of which requires substantial support from institutional leadership, ensuring that there is alignment and commitment.

In the evaluation of the JHI model and resources required to support the model and JHI portfolio of projects, we found that significant human resources are required within a broad range of AMC subject matter expertise across multiple disciplines. For AMCs considering expanding their services or network, consideration should be given to the model, resources and requirements to ensure success and that with forecasting, AMCs can engage in these collaborations abroad while continuing to fulfill their core mission at home.
